# Consumer trait variation influences tritrophic interactions in salt marsh communities

**DOI:** 10.1002/ece3.1564

**Published:** 2015-06-17

**Authors:** Anne Randall Hughes, Torrance C Hanley, Nohelia P Orozco, Robyn A Zerebecki

**Affiliations:** 1 Marine Science Center, Northeastern UniversityNahant, Massachusetts; 2 Coastal and Marine Laboratory, Florida State UniversitySt. Teresa, Florida

**Keywords:** Behavior, foundation species, host plant, individual specialization, intraspecific variation, microgeographic divergence, phenotypic plasticity, preference induction, trophic cascade

## Abstract

The importance of intraspecific variation has emerged as a key question in community ecology, helping to bridge the gap between ecology and evolution. Although much of this work has focused on plant species, recent syntheses have highlighted the prevalence and potential importance of morphological, behavioral, and life history variation within animals for ecological and evolutionary processes. Many small-bodied consumers live on the plant that they consume, often resulting in host plant-associated trait variation within and across consumer species. Given the central position of consumer species within tritrophic food webs, such consumer trait variation may play a particularly important role in mediating trophic dynamics, including trophic cascades. In this study, we used a series of field surveys and laboratory experiments to document intraspecific trait variation in a key consumer species, the marsh periwinkle *Littoraria irrorata*, based on its host plant species (*Spartina alterniflora* or *Juncus roemerianus*) in a mixed species assemblage. We then conducted a 12-week mesocosm experiment to examine the effects of *Littoraria* trait variation on plant community structure and dynamics in a tritrophic salt marsh food web. *Littoraria* from different host plant species varied across a suite of morphological and behavioral traits. These consumer trait differences interacted with plant community composition and predator presence to affect overall plant stem height, as well as differentially alter the density and biomass of the two key plant species in this system. Whether due to genetic differences or phenotypic plasticity, trait differences between consumer types had significant ecological consequences for the tritrophic marsh food web over seasonal time scales. By altering the cascading effects of the top predator on plant community structure and dynamics, consumer differences may generate a feedback over longer time scales, which in turn influences the degree of trait divergence in subsequent consumer populations.

## Introduction

Intraspecific variation, whether genetic, environmental, or developmental, can have significant effects on community structure and ecosystem function (Bolnick et al. 2003, 2011; Whitham et al. 2006; Hughes et al. 2008). Although much of this research has focused on plants, there is also substantial evidence for important effects of consumer trait differences, both at intermediate and top trophic levels (Bolnick et al. 2003, 2011). For example, trait variation within top predator species (e.g., body size variation: Werner and Gilliam 1984; Miller and Rudolf 2011; functional trait variation: Zhao et al. 2014) can influence predator–prey interactions and the strength of top-down control (Osenberg and Mittelbach 1989; Delclos and Rudolf 2011; Rudolf 2012). Further, intraspecific phenotypic differences in an intermediate planktivorous fish species affect zooplankton and phytoplankton community structure, as well as the strength of lake trophic cascades (Post et al. 2008; Palkovacs and Post 2009; Walsh et al. 2012). The central position of consumer species within a tritrophic system (Trussell and Schmitz 2012) means that consumer intraspecific diversity, in particular, has the potential to mediate cascading effects on community and ecosystem processes (e.g., Gamfeldt et al. 2005; Agashe 2009; Ellers et al. 2011; Griffen et al. 2012). Such differences may be particularly likely when the consumer trophic level is species-poor (i.e., the consumer species has few competitors; Dall et al. 2012).

Many small-bodied consumers such as insects and marine invertebrates live on host plants that provide both nutrition and habitat (Price et al. 1980; Strong et al. 1984; Hay et al. 1987; Duffy and Hay 1991; Singer et al. 2004). This coupling can result in trait differences within and among consumer species on particular host plant species (Price et al. 1980; Hay et al. 1987; Richardson et al. 2014) and may reflect differences in host plant quality as a food source, a predation refuge, or both. This association may also impact predator–prey interactions, potentially altering the strength of top predator effects in a food web (Singer et al. 2014). The interdependence of consumers on their host plants for both food and refuge highlights the need for a tritrophic approach to understand the role of host plant-associated consumer trait variation on the direction and magnitude of trophic cascades and the consequent effects on population dynamics and community structure (Singer et al. 2004). Variation at the consumer level, including genetic diversity and phenotypic plasticity, is predicted to be more stabilizing than variation at the producer level alone or across multiple trophic levels (i.e., both producer and consumer levels; Kovach-Orr and Fussman 2013). Thus, understanding the effects of consumer trait variation in a tritrophic context has important implications for the long-term dynamics of populations, communities, and ecosystems in this era of rapid environmental change.

**Figure 1:**
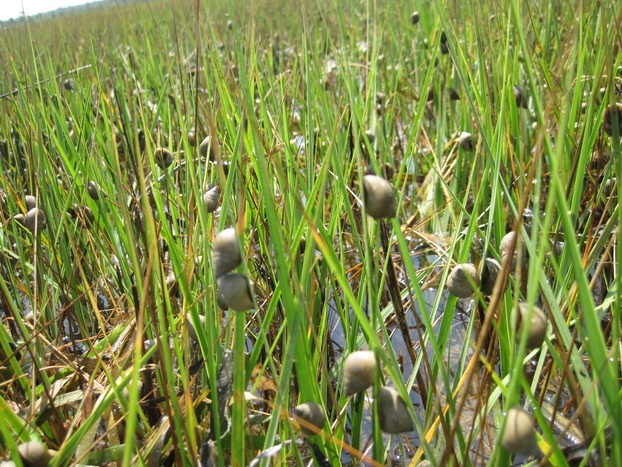
The marsh periwinkle *Littoraria irrorata*, a common consumer species in salt marshes of the Gulf of Mexico and Southeastern USA, climbing on marsh cordgrass *Spartina alterniflora*.

We used a tritrophic perspective to investigate the extent and effects of host plant-associated consumer trait variation on plant species interactions, biomass, and community structure in the presence and absence of a top predator. Predator presence can have strong effects on consumer morphology and behavior (Preisser et al. 2005; Preisser and Bolnick 2008), even leading to increased specialization among prey (Araujo et al. 2011). Thus, we hypothesized that consumer behavioral trait variation (e.g., feeding and climbing behavior) may be magnified in the presence of predators, with potential cascading effects on plants. We examined these interactions in salt marsh communities – tritrophic systems in which little attention has been given to the role of consumer trait variation (but see Atkins et al. 2015) – which typically include competitive and facilitative plant–plant interactions (Hughes 2012), strong consumer control (Bertness and Silliman 2008; Long et al. 2011; Altieri et al. 2012; Daleo et al. 2014), and cascading effects of top predators (Silliman and Bertness 2002; Kimbro 2012; Bertness et al. 2014). First, we assessed the presence and magnitude of intraspecific variation in morphological and behavioral traits, isotopic composition, and predation susceptibility in the snail consumer *Littoraria irrorata* (Fig.1) based on the host plant species it utilizes in the field (*Spartina alterniflora* or *Juncus roemerianus*). We then conducted a mesocosm experiment to examine the ecological effects of snail consumer trait variation in the presence and absence of a gastropod predator (crown conch, *Melongena corona*) that elicits escape behavior in *Littoraria* (Dix and Hamilton 1993).

## Materials and Methods

### Study system

Salt marshes provide an ideal system for testing the effects of intermediate trophic level variation on tritrophic interactions. Salt marsh communities are typically comprised of a few strongly interacting plant species that vary in competitive ability across gradients in nutrient availability and environmental stress (Bertness and Ellison 1987; Pennings and Bertness 2001; Pennings et al. 2002). Although traditionally considered unimportant, consumer effects can be strong in salt marsh communities and ecosystems (Bertness and Silliman 2008; Long et al. 2011; Altieri et al. 2012; Daleo et al. 2014), including cascading effects of top predator presence and identity on consumers and plants (Silliman and Bertness 2002; Kimbro 2012; Bertness et al. 2014). Finally, salt marshes are ecologically and economically valuable ecosystems (Barbier et al. 2011) that have experienced significant declines worldwide (Lotze et al. 2006); thus, insights regarding the effects of consumer variation and predator presence on plant community structure and productivity have implications for both conservation and restoration.

We focus here on the marsh consumer *Littoraria irrorata*, a common inhabitant of Gulf of Mexico and southeast Atlantic salt marshes, that is the primary consumer of the dominant plant species, *Spartina alterniflora* (Silliman et al. 2005; Hughes 2012; Kimbro 2012). This snail uses *Spartina* both as a source of food and as a refuge from marine predators by climbing up the plant stems at high tide (Hughes 2012*). Littoraria* was long considered a detritivore, but it exhibits a range of feeding strategies, including consuming detritus and live *Spartina*, and farming fungus in grazing wounds on live *Spartina* stems (Silliman and Newell 2003). Although most studies of *Littoraria* have focused on its relationship with *Spartina*, snails are commonly found on both *Spartina* and *Juncus roemerianus* in marshes where these plant species codominate in the northern Gulf of Mexico (Hughes 2012). In natural assemblages where the plant species co-occur at scales of 1–3 m^2^, snails of either type are likely to encounter the alternative host plant species over daily or weekly time scales, and preliminary observations indicated trait differences between the snails collected from each host plant species in these mixed assemblages (see Results). Thus, we quantified variation in traits between the snails collected from *Spartina* (hereafter, S-snails) and the snails collected from *Juncus* (hereafter, J-snails) in mixed plant assemblages. We then conducted a mesocosm experiment to examine whether snail type differentially impacted plant assemblages with *Spartina* only, *Juncus* only, or *Spartina* and *Juncus* mixed together in the presence and absence of the predatory crown conch *Melongena corona*.

This study was conducted in Apalachee Bay and St. Joseph Bay, FL. Much of the shoreline in this region of the northeastern Gulf of Mexico is bordered by salt marsh habitat, including single species and mixed stands of *Spartina alterniflora* and *Juncus roemerianus* (Hughes 2012). The consumer *Littoraria irrorata* and the predator *Melongena corona* are common in all of these plant assemblages, both in our study region and the broader Gulf of Mexico (Hayes 2003; Silliman et al. 2005; Hughes 2012; Zerebecki and Hughes 2013). *Littoraria* utilizes both *Spartina* and *Juncus* as a predation refuge: by climbing up standing live and dead stems, snails can escape from benthic predators including *Melongena* and the blue crab *Callinectes sapidus* (Hughes 2012). Mesocosm experiments were conducted at the Florida State University Coastal and Marine Laboratory (FSUCML).

### Trait differences between snail types

To assess the morphological differences between S-snails and J-snails, *Littoraria* were collected from *Spartina* and *Juncus* host plants (*N* = 28 per host plant species) separated by 1–3 m in a mixed plant assemblage in St. Joseph Bay, FL. *Littoraria* shell length and width, aperture length and width, and mean ridge thickness (estimate of shell thickness, calculated as the average of central, anterior, and posterior thickness; see fig. 1 in Moody and Aronson (2012)) were measured using digital calipers.

Controlled feeding assays were conducted to quantify S-snail and J-snail consumption of *Spartina* and *Juncus* ground tissue. Snails were collected from each host plant (*N* = 300 per species) within a mixed plant assemblage in Apalachee Bay, FL. The snails were housed in the laboratory and fed seagrass detritus and spritzed with saltwater every 2 days. *Spartina* and *Juncus* stems were also collected from the same field site, omitting any that had multiple *Littoraria* grazing scars. The plant stems were sealed in separate plastic bags with kitty litter for approximately 1 month to extract moisture from the tissue, ground to a fine consistency using a plant mill, and then incorporated into agar using the methods of Long et al. (2011). Once both plant tissue samples had been prepared and allowed to cool, one dish of agar from each plant species was placed in a plastic container with no holes to prevent the tissue from drying out. We haphazardly selected four J-snails or S-snails, measured the shell length of each, and then added them to each container with 2 mL of water for 4 days, or until half of the tissue samples had been consumed. We ran 43 replicates for each snail type across three trials (one trial per week; *N* = 12 per snail type in trials 1 and 2; *N* = 19 per snail type in trial 3). At the end of the trial, window screen was placed underneath each dish and the number of squares of agar of each plant species that had been consumed was counted.

To assess the potential for diet variation between S-snails and J-snails, we used stable isotope analysis (stable carbon isotope ratios δ^13^C and stable nitrogen isotope ratios δ^15^N). Stable isotopes integrate diet composition over time scales of weeks to months (Post 2002; Snowberg et al. 2015), and thus, they can also provide information regarding host plant fidelity in S-snails and J-snails. Because stable C isotopes differentiate between C3 (i.e., *Juncus roemerianus*) and C4 (i.e., *Spartina alterniflora*) plants (Fry and Sherr 1989), we measured δ^13^C and δ^15^N for 20 S-snails and 18 J-snails to characterize differences in isotopic dietary niche between snail types. Tissue from each snail was dried at 60°C for 48 h, ground using a mixer mill MM400 (Retsch), and then 0.9–1.1 mg of each sample was weighed into a tin capsule (Costech) for analysis on a Costech ECS 4010 elemental analyzer interfaced to a Thermo Delta^Plus^ Advantage mass spectrometer (Thermo Finnigan) at the Yale Analytical and Stable Isotopic Center.

The susceptibility of S-snails and J-snails to *Melongena* was assessed in the laboratory. S-snail and J-snail susceptibility was compared separately for both live and dead snails to assess both predator preference and the potential influence of snail behavior on consumption. Snails were collected from salt marshes in St. Joseph Bay and Apalachee Bay, FL, and were housed as described in the feeding trials above. S-snails and J-snails were painted with different colored enamel prior to each assay to distinguish between them. Five S-snails and J-snails were placed in each mesocosm (*N* = 10) with a single crown conch in a flow-through water table at the FSUCML greenhouse. Mesocosms did not include plants; snails and predators were allowed to move freely around the mesocosms, including climbing the sides. Trials with live snails were conducted in February 2010 for 5 weeks as a replacement design. Mesocosms were checked weekly, consumed snails were measured and recorded, and consumed S-snails and J-snails were replaced. Trials with dead snails were conducted in July 2014 to examine predator preference in the absence of snail behavior. Prior to the experiment, snails were collected from the field and frozen (−80^°^C) for 24 h. Trials were conducted over 24 h to prevent the decay of snail tissue from obscuring predation events. We ran a total of 21 replicates across four trials (*N* = 5 replicates per trial for trials 1–3; *N* = 6 replicates for trial 4).

### Effects of snail trait variation in the presence and absence of a predator

A mesocosm experiment was conducted in summer 2012 to examine the effects of snail trait variation on tritrophic interactions in *Spartina*-only, *Juncus*-only, and mixed plant assemblages. All possible combinations of plant treatment (*Juncus* only, *Spartina* only, or *Juncus–Spartina* mix), snail treatment (S-snails vs. J-snails), and predator treatment (crown conch present vs. absent) were tested. All plants and snails were collected from salt marshes in St. Joseph Bay and Apalachee Bay, Florida. Snails (*N* = 800) were collected from each host plant within a mixed marsh assemblage in May 2012. At the same time, 80 clumps of *Juncus* with six to eight stems were collected from the field and planted in separate flower pots in a greenhouse at the Florida State University Coastal and Marine Laboratory (FSUCML). Six different *Spartina* genotypes collected from natural marshes in July 2009 and propagated in the greenhouse at the FSUCML were also used (Hughes et al. 2014).

We used 72 flow-through seawater mesocosms (volume = 5.19 L) in this experiment. Mesocosms were grouped within 1.5-m diameter pools (6 mesocosms per pool) to facilitate drainage. Mesocosms were filled with sieved sand to a height of 25 cm, with three holes in the bottom to allow for drainage. Rectangular cages consisting of a PVC frame covered with mesh netting (*H* = 74.7 cm; *W* = 30.5 cm; *L* = 30.5 cm; volume=10.75 L) were placed in each mesocosm to contain the snails. Treatments were assigned at random to the six mesocosms within two adjacent pools; these two pools were treated as a statistical block. We added either two Spartina transplants (mean [SE] *Spartina* live stem density per transplant = 7.7 [0.33]), two Juncus transplants (mean [SE] *Juncus* live stem density per transplant = 4.13 [0.25]), or one Spartina and one Juncus transplant to mesocosms 2 weeks prior to starting the experiment to allow them to acclimate to the experimental setting. During this period, plants were watered everyday with freshwater. At the end of the 2-week acclimation period, the number of live and dead stems of each plant species and the height of each live stem were recorded. Stem height and density often predict plant competition intensity (Cahill et al. 2008), influence plant–plant (Emery et al. 2001) and plant–consumer interactions (Hughes 2012; Zerebecki and Hughes 2013), and determine the extent of important ecosystem services such as wave attenuation and shore stabilization provided by salt marshes (Shepard et al. 2011). Fifteen adult J-snails or S-snails were added to each mesocosm, which is within the range of naturally observed densities in this system (Hughes 2012). Potential effects of differences in snail size between J-snails and S-snails were minimized using a representative standardized distribution in snail shell length across all mesocosms: three snails measuring 15–16 mm, nine snails measuring 17–18 mm, and three snails measuring 19–20 mm. One predatory crown conch (*Melongena corona*) was added into each predator treatment a day after introducing the snails. During the 12-week experiment, a diurnal tidal regime was simulated by submerging the plants with flow-through seawater every day for 6 h. Each mesocosm was also watered with freshwater three times a week for 5 min.

To examine the potential differences in snail climbing behavior, the number of snails climbing on each plant species or the mesocosm walls at both low and high tide was counted at approximately weekly intervals (*N* = 10 times over the 12-week experiment). The average number of snails climbing on plants (regardless of plant species) over the duration of the experiment was calculated as a measure of snail climbing behavior at both low and high tide. To examine the relationship between snail climbing behavior and plant responses, the average number of snails climbing per plant stem of each plant species (*Spartina* and *Juncus*) was then calculated at both low and high tide.

At the end of twelve weeks, the number of live and dead stems and the height of all live stems were measured. In addition, the number of live and dead snails was counted in each mesocosm. The plants were harvested and divided into aboveground and belowground biomass by plant species. The plant tissue was dried for at least 48 h at 60°C before the dry weight was recorded.

### Statistical analyses

To assess the differences in morphological traits and allometry between J-snails and S-snails, we first conducted a two-tailed, unpaired *t*-test for shell length (i.e., snail size) to test for differences in shell length between snail types. We then used analysis of covariance (ANCOVA) to compare the relationship between shell length (covariate) and each additional shape variable (shell width, ridge thickness, and aperture length, width, and ratio) for each snail type (fixed factor).

Differences in consumer feeding behavior (total plant material consumed) were assessed using a mixed-effect generalized linear model (GLM) using the lme4 package in R version 3.0.2, with a fixed effect of snail type, a random effect of trial, a random effect of container, and average snail shell length per container as a covariate. We also included the interaction between snail type and average shell length. For all GLMs, the Satterthwaite approximation for degrees of freedom from the lmerTest package was used to generate F and *P*-values, followed by Tukey’s post hoc mean comparisons using the multcomp package. Finally, paired *t*-tests were used to examine the susceptibility of S-snails and J-snails to crown conchs in both the live and dead snail experiments.

To assess the differences in isotopic composition and diet between S-snails and J-snails collected in the field, we first performed a multivariate analysis of variance (MANOVA) on stable C and N isotope ratios. Because the MANOVA identified significant differences in isotope ratios between S-snails and J-snails (see Results), a two-tailed, unpaired *t*-test was conducted for each isotopic measurement to determine which component(s) differed between snail types.

For the mesocosm experiment, a series of mixed-effect GLMs using the lme4 package were conducted including a random effect of block and all possible interactions among fixed effects of plant treatment (*Spartina*-only, *Juncus*-only, and mixed), snail type (J-snails or S-snails), and predator treatment (present or absent). Variation in snail climbing behavior was examined at both low and high tide over the course of the experiment using the average number of snails climbing on plants across all sampling dates. The number of snails climbing on each plant species was then analyzed separately to examine the relationship between snail climbing behavior and plant responses. To account for variation in snail abundance due to predation or other mortality, the number of dead snails at the end of the experiment was used as a covariate in these analyses. Plant responses, including total plant stem density, average stem height, aboveground plant biomass, and belowground plant biomass were also examined. To compare more accurately across plant treatments, an effect size metric for both stem density and stem height was calculated as the difference between final and initial values standardized by initial values. There were no initial values for aboveground or belowground biomass, so these analyses were run on the final values only.

To look more closely at individual plant species responses, plant density, average height, and aboveground and belowground biomass were examined by plant species in the plant treatments in which they occurred (i.e., *Juncus* density in the *Juncus*-only and mixed plant treatments). For the analyses of stem density and height, the effect size metrics were used as above.

## Results

### Trait differences among snails

**Figure 2:**
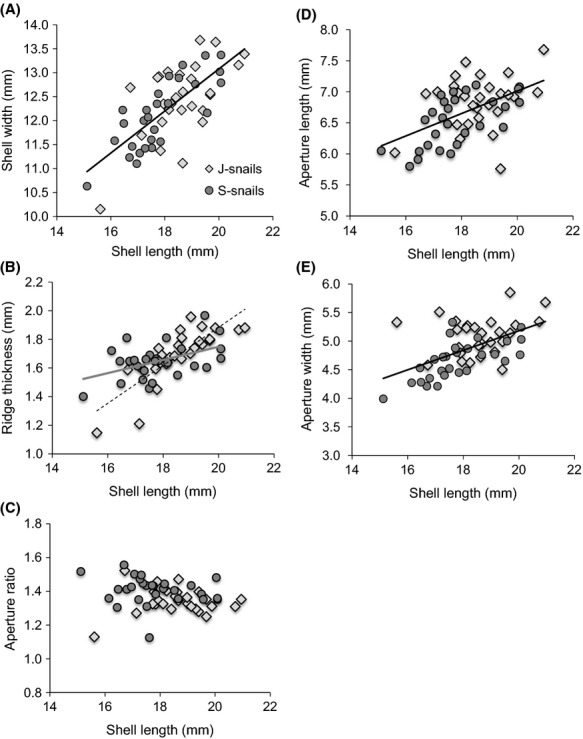
Morphological differences between types of the snail *Littoraria irrorata*. J-snails (light gray diamonds) were collected from the host plant *Juncus roemerianus*; S-snails (dark gray circles) were collected from the host plant *Spartina alterniflora*. If there was a significant difference in slope between snail types, the relationship between each morphological trait and shell size is represented by solid gray and dashed black lines for S-snails and J-snails, respectively; if there was no significant difference in slope between snail types, the relationship is represented by a solid black line. The relationship between snail shell length in mm and (A) shell width in mm (S-snails and J-snails: *y* = 0.44*x *+ 4.30, *R*^2^ = 0.52, *P* < 0.001); (B) mean ridge thickness in mm (J-snail: *y* = 0.13*x* − 0.77, *R*^2^ = 0.66, *P* < 0.001; S-snail: *y* = 0.05*x *+ 0.83, *R*^2^ = 0.22, *P* = 0.01); (C) aperture ratio (length:width) (S-snails and J-snails: *y* = −0.01*x *+ 1.61, *R*^2^ = 0.04, *P* = 0.14). (D) aperture length in mm (S-snails and J-snails: *y* = 0.18*x *+ 3.40, *R*^2^ = 0.27, *P* < 0.001). (E) aperture width in mm (S-snails and J-snails: *y* = 0.17*x *+ 1.77, *R*^2^ = 0.30, *P* < 0.001). Shell characteristics defined as in Moody and Aronson (2012) and Bourdeau (2009).

J-snails had larger shells than S-snails (shell length *t*-test *P* = 0.019; mean ± SE: J-snails 18.60 mm ± 0.22; S-snails 17.81 mm ± 0.24). In addition, the allometric relationship between ridge thickness (calculated as the average of the anterior, central, and posterior thickness; Moody and Aronson 2012) and shell length differed significantly between snail types (ANCOVA: *F*_1,51_ = 11.65, *P* = 0.001; Fig.2B). However, the slope of the relationship between shell length and other shell morphological characteristics (e.g., shell width, aperture ratio, aperture length, aperture width) did not differ between snail types (Fig.2), suggesting that most morphological differences across snail types are due to differences in overall size.

**Figure 3:**
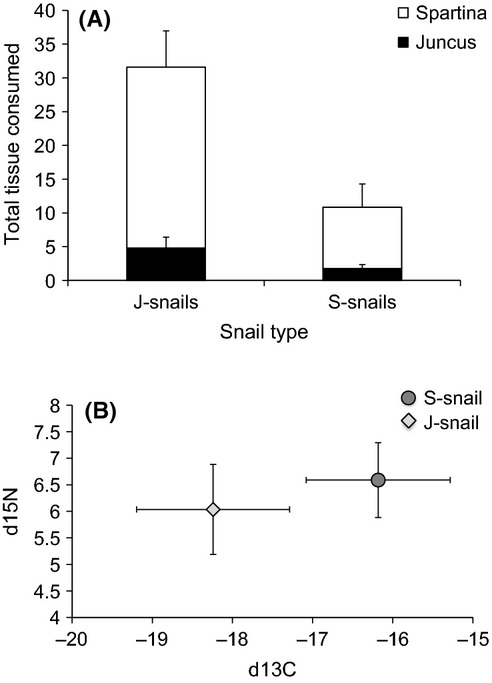
Feeding differences between types of the snail *Littoraria irrorata*. J-snails were collected from the host plant *Juncus roemerianus*; S-snails were collected from the host plant *Spartina alterniflora*. The total number of squares of agar-based food consumed by each snail type in a choice feeding trial with *Spartina*-based agar and *Juncus*-based agar. Consumption of *Spartina* tissue is indicated by the open portion of the bars; consumption of *Juncus* tissue is indicated by the black portion of the bars. Error bars represent + 1SE. (B) Isotopic composition of S-snails and J-snails collected from the field; light gray diamonds and dark gray circles represent mean ± SD values of δ^13^C and δ^15^N for J-snails and S-snails, respectively.

In controlled feeding trials, there was a significant effect of snail type (*F*_1,81 _= 3.99, *P* = 0.049; Fig.3A), with J-snails exhibiting greater consumption of the agar-based food than S-snails. This difference was primarily due to higher consumption of the *Spartina*-based agar rather than the *Juncus*-based agar (Fig.3A). Although the J-snails used in our trials were larger on average than the S-snails (*F*_1,81 _= 21.45, *P* < 0.001), neither average snail length (*F*_1,81 _= 1.94, *P* = 0.17) nor the interaction between snail length and snail type (*F*_1,81 _= 0.57, *P* = 0.45) were significant predictors of snail consumption.

The isotopic composition of S-snails and J-snails collected from mixed plant communities differed significantly (MANOVA snail type *F*_1,35_ = 23.09, Pillai’s trace = 0.57, *P* < 0.001; Fig.3B). The snail types had distinct δ^13^C values (*t*-test *P* < 0.001), with δ^13^C of S-snails (mean = −16.2‰) potentially reflecting a greater proportion of *Spartina* (−13‰; Sullivan and Moncreiff 1990) in their diet and δ^13^C of J-snails (mean = −18.2‰) potentially reflecting a greater proportion of *Juncus* (−26‰; Sullivan and Moncreiff 1990) in their diet. In addition, δ^15^N of snail types differed significantly (*t*-test *P* = 0.03), with S-snails having higher δ^15^N values than J-snails.

**Figure 4:**
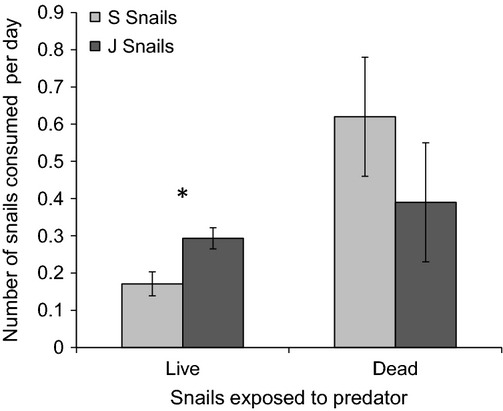
Susceptibility of *Littoraria irrorata* snail types to predation by the crown conch *Melongena corona*. Number of snails consumed per day in separate experiments with live snails and dead snails. Bars represent means ± 1SE. *Indicates significant differences at *P* < 0.05.

When exposed to predators, more live J-snails were consumed than live S-snails (*t*-test *P* = 0.02; Fig.4), yet there was no difference in consumption of the two snail types when snails were dead (*t*-test *P* = 0.31; Fig.4). The average size of snails consumed did not differ between S-snails and J-snails for either trial (live snail *t*-test *P* = 0.61; dead snail *t*-test *P* = 0.22). However, in the dead snail susceptibility trials, unconsumed *Spartina* snails were smaller (17.27 mm ± 0.13) than unconsumed *Juncus* snails (17.73 mm ± 0.13, *t*-test *P* = 0.02).

**Figure 5:**
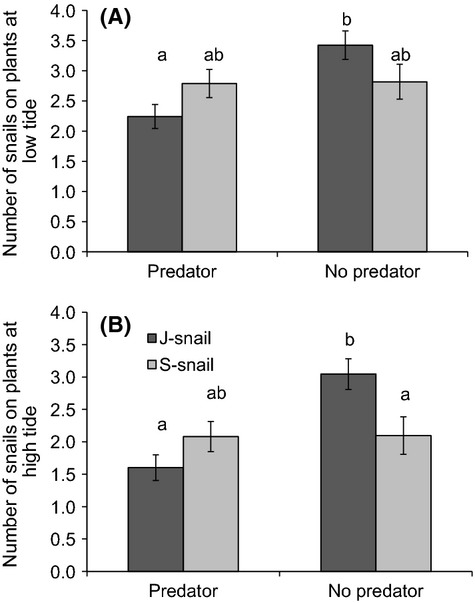
Variation in snail climbing behavior at (A) low tide and (B) high tide in experimental mesocosms. The number of snails climbing up plant stems varied depending on predator presense/absence and snail type. Letters indicate significant differences at *P* < 0.05 based on Tukey’s post hoc tests. Bars represent means ± 1SE.

J-snails and S-snails also differed in climbing behavior over the course of our mesocosm experiment, and this response varied in the presence and absence of a predator: at low tide, more J-snails were on plants in the absence of a predator than in its presence, whereas S-snails showed no difference (snail*predator *F*_1,54 _= 5.72, *P* = 0.02; Fig.5A). The effects of predators and snail trait variation on snail climbing behavior were similar at high tide (snail*predator *F*_1,59 _= 7.06, *P* = 0.01; Fig.5B). There was also an independent effect of plant treatment at high tide (plant *F*_2,59 _= 4.28, *P* = 0.02), with more snails on plants in the *Juncus*-only and mixed treatments than in *Spartina*-only. Not surprisingly, there was a greater number of dead snails when predators were present (mean [SE] = 2.67 [0.35]; predator *F*_1,59 _= 22.78, *P* < 0.001) than when they were absent (mean [SE] = 0.75 [0.17]), but there was no effect of plant treatment or snail type.

### Effects of snail trait variation on plant communities in a tritrophic system

**Figure 6:**
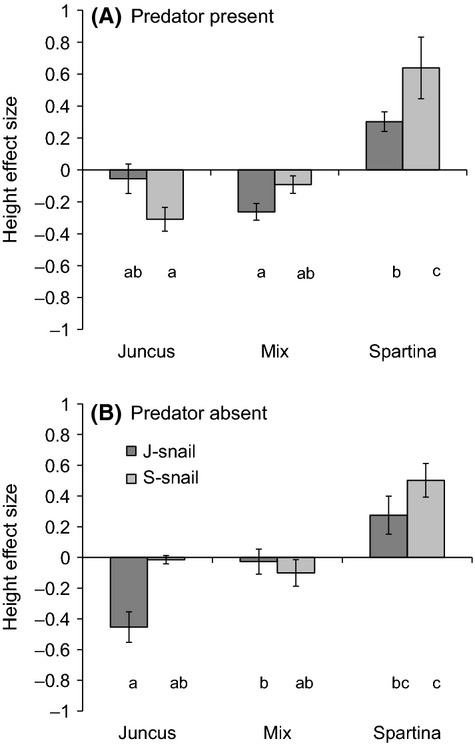
Effects of plant community composition and snail type on the overall change in plant stem height in the (A) presence and (B) absence of a predator over the course of a 12-week mesocosm experiment. (A) When predators were present, the effects of snails differed by snail type in the *Spartina*-only community. (B) When predators were absent, the differential effects of the two snail types were reduced. In addition, the negative effect of J-snails on plant height in the presence of predators disappeared when predators were absent in the mixed community. Letters indicate significant differences at *P* < 0.05 based on Tukey’s post hoc tests. Bars represent means ± 1SE.

**Figure 7:**
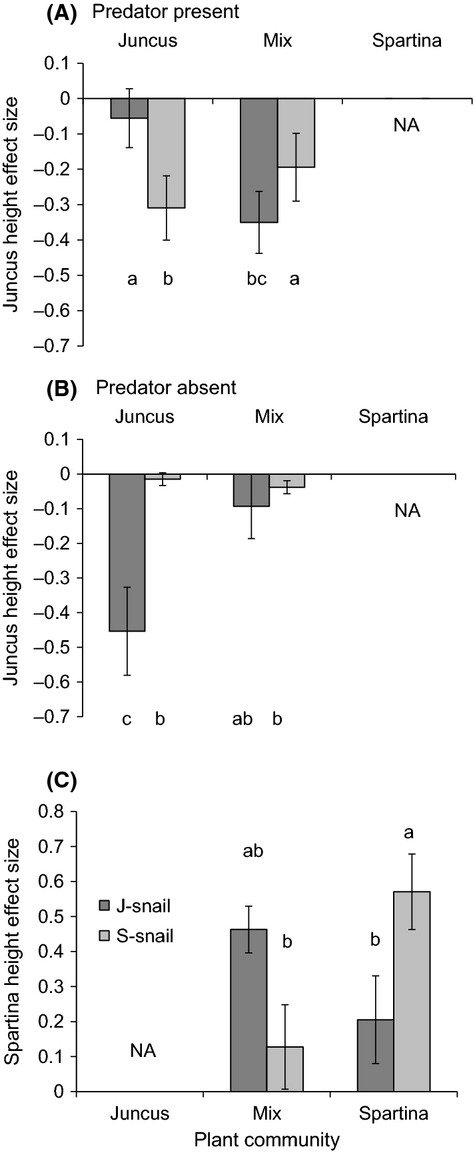
Effects of plant community composition, snail type, and predator presence on the change in (a,b) *Juncus* stem height and (c) *Spartina* stem height over the course of a 12-week mesocosm experiment. (A) When predators were present, S-snails caused a reduction in *Juncus* stem height in both plant communities where *Juncus* was present. In contrast, J-snails only caused a reduction in stem height in the mixed community. (B) When predators were absent, J-snails caused a reduction in *Juncus* stem height in the *Juncus*-only but not the mixed community. S-snails had minor effects on *Juncus* stem height that did not differ by plant community. (C) Changes in *Spartina* stem height varied by plant community composition and snail type, but they were consistent across predator treatments. Letters indicate significant differences at *P* < 0.05 based on Tukey’s post hoc tests. Bars represent means ± 1SE.

Snail trait variation and predator presence/absence led to differences in overall plant height across plant treatments (plant*predator*snail *F*_2,52 _= 7.05, *P* = 0.002; Fig.6, Table1): the differences between J-snails and S-snails were greatest in *Spartina*-only communities in the presence of predators (Fig.6A), consistent with a stronger predator avoidance response of S-snails. Overall, plant height decreased in *Juncus*-only plant communities and increased in *Spartina*-only plant communities, with slight decreases to no change in mixed plant communities (Fig.6). The differences in overall plant height are due to snail effects on the height of both *Juncus* and *Spartina* (Fig.7). In *Juncus*-only communities, J-snails had strong negative effects on plant height in the absence of predators, whereas S-snails had negative effects on *Juncus* height only in the presence of predators (plant*predator*snail *F*_1,39_ = 4.72, *P* = 0.04; Fig.7A and B). In mixed plant communities, J-snails had stronger negative effects on *Juncus* height than S-snails when predators were present, but both snail types had equivalent and negligible effects in the absence of predators (Fig.7A and B). J-snails and S-snails also differentially affected *Spartina* height across plant treatments (plant*snail *F*_1,40 _= 10.08, *P* = 0.003; Fig.7C): *Spartina* stem height increased more in the presence of J-snails than S-snails in mixed communities, but stem height increased more in the presence of S-snails than J-snails in *Spartina*-only communities. These effects on *Spartina* were consistent regardless of predator presence or absence.

**Table 1:** Results of statistical analyses for response of total stem height, total stem density, total plant aboveground biomass, and total plant belowground biomass to plant treatment, snail type, and predator treatment

Factor	Num df	Stem height (Den df = 52)		Stem density (Den df = 55)		Above biomass (Den df = 48)		Below biomass (Den df = 52)	
		*F*	*P*	*F*	*P*	*F*	*P*	*F*	*P*
Plant treatment	2	48.83	**<0.001**	12.60	**<0.001**	10.69	**<0.001**	12.50	**<0.001**
Snail type	1	4.55	**0.037**	3.14	*0.082*	0.07	0.793	0.63	0.431
Predator	1	0.01	0.917	5.26	**0.025**	4.38	**0.041**	0.07	0.787
Plant treatment ^*^ Snail type	2	1.16	0.321	0.37	0.690	0.75	0.475	0.12	0.891
Plant treatment ^*^ Predator	2	0.82	0.447	1.44	0.244	0.22	0.801	0.07	0.935
Snail type ^*^ Predator	1	1.09	0.301	0.64	0.428	0.62	0.433	0.17	0.681
Plant treatment ^*^ Snail type ^*^ Predator	2	7.05	**0.002**	1.72	0.189	1.90	0.160	0.15	0.859

Bold indicates significant at *P* < 0.05; Italics indicates marginally significant at *P* < 0.10.

Snail type had significant effects not only on *Juncus* stem height, but also on *Juncus* stem density and aboveground biomass (Fig. S1). S-snails caused a greater reduction in the number of live *Juncus* stems than J-snails (snail *F*_1,40 _= 4.06, *P* = 0.05; Fig. S1a). In addition, more *Juncus* stems were lost in the *Juncus*-only compared to the mixed plant treatments (plant *F*_1,40 _= 8.53, *P* = 0.006; Fig. S1a), suggesting that the presence of *Spartina* provided a benefit to *Juncus* in this experiment. The effects of J-snails and S-snails varied interactively across plant and predator treatments for *Juncus* aboveground biomass in a manner similar to *Juncus* height (plant*predator*snail *F*_1,26_ = 6.99, *P* = 0.01). J-snails had a more negative effect on *Juncus* biomass in mixed communities compared to *Juncus*-only communities in the presence of predators (Fig. S1b,c), yet the opposite pattern occurred in the absence of predators, in that J-snails had a greater negative effect on *Juncus* biomass in *Juncus*-only communities (Fig. S1b,c). S-snails had a greater negative effect on *Juncus* biomass in *Juncus*-only communities than mixed communities in the presence of predators, but the effects of S-snails on *Juncus* biomass did not vary by plant treatment in the absence of predators (Fig. S1b,c).

Plant treatment and predator treatment independently affected both total plant stem density (plant *F*_2,55 _= 12.60, *P* < 0.001; predator *F*_1,55 _= 5.26, *P* = 0.03; Fig. S2, Table1) and final plant aboveground biomass (plant *F*_2,48 _= 10.69, *P* < 0.001; predator *F*_1,48 _= 4.38, *P* = 0.04; Fig. S2b, Table1). Belowground biomass differed by plant treatment only, with higher biomass in *Spartina*-only and mixed plant communities than in *Juncus*-only communities (plant *F*_2,52 _= 12.50, *P* < 0.001). There was also a marginal effect (*F*_1,55 _= 3.14, *P* = 0.08) of snail treatment on the total number of live stems, with S-snails having a greater negative effect (mean [SE] effect size = −0.46 [0.06]) on live stem density than J-snails (mean [SE] effect size = −0.32 [0.07]).

Snail type did not influence *Spartina* density or aboveground biomass. Rather, *Spartina* density was affected by predator treatment (predator *F*_1,40 _= 4.09, *P* = 0.05): There were more live *Spartina* stems remaining when predators were present (mean [SE] effect size = −0.10 [0.09]) than absent (mean [SE] effect size = −0.34 [0.13]). *Spartina* aboveground biomass was higher in *Spartina*-only communities (plant *F*_1,33_ = 8.10, *P* = 0.007) and marginally higher in the presence of predators (predator *F*_1,33 _= 3.10, *P* = 0.09; Fig. S2c).

**Figure 8:**
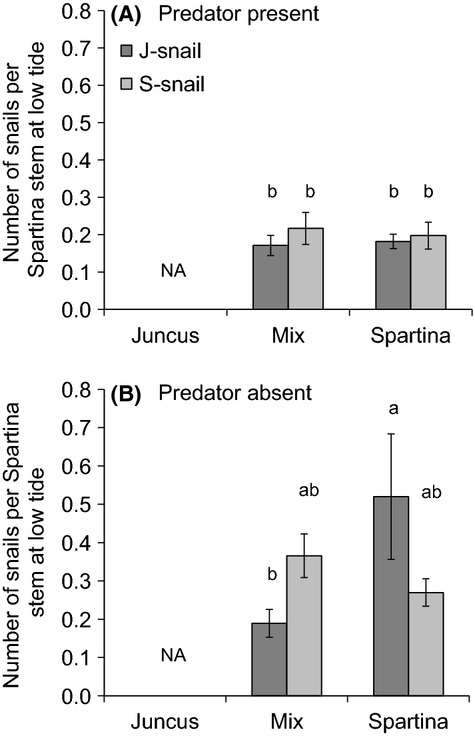
Number of snails of each type per *Spartina* stem at low tide in the (A) presence and (B) absence of a predator in a 12-week mesocosm experiment. The number of snails per *Spartina* stem was equivalently low across snail types and plant communities in the presence of a predator. In contrast, more J-snails climbed on *Spartina* in the *Spartina*-only treatment than in the mixed community when predators were absent. Letters indicate significant differences at *P* < 0.05 based on Tukey’s post hoc tests. Bars represent means ± 1SE.

Snail climbing behavior was a weak predictor of plant responses in our mesocosm experiment. The number of snails climbing per *Spartina* stem at low tide varied by snail type, plant treatment, and predator treatment (plant*predator*snail *F*_1,35 _= 4.31, *P* = 0.04; Fig.8), with more S-snails than J-snails on *Spartina* in mixed plant communities and more J-snails than S-snails on *Spartina* in *Spartina*-only communities in the absence of a predator (Fig.8B). These patterns in snail behavior were consistent at high tide (Fig. S3) and mirrored the differential effects of snail types on *Spartina* height (Fig.7C). There were also more snails climbing per plant on *Juncus* stems (0.53 per stem) than *Spartina* stems (0.12 per stem) over the course of the experiment (*t*-test *P* < 0.001), consistent with the overall negative effects of snails on *Juncus*. Yet despite the fact that the number of snails climbing on *Juncus* was consistent across plant, snail type, and predator treatments, the response of *Juncus* varied across these same treatments (Fig.7, S1).

## Discussion

*Littoraria* exhibited significant variation in a range of key traits, including morphology, feeding behavior, isotopic composition, and climbing behavior, depending on its host plant species (i.e., the plant it is collected from in the field: *Spartina* or *Juncus*). These differences occurred among snails separated by only 1–3 m in natural mixed plant assemblages, and they persisted for several months in our experimental common garden setting, with cascading effects on the plant community. Thus, our data contribute to the growing literature on microgeographic divergence, or trait differences that occur across fine spatial scales (Richardson et al. 2014; Langin et al. 2015).

J-snails and S-snails also differed in their response to a common snail predator, the crown conch (*Melongena corona*): J-snails were less likely to climb on plants when crown conchs were present in our mesocosm experiment, whereas S-snails were equally likely to climb on plants in both the presence and absence of conchs. Further, crown conchs preferentially consumed J-snails over S-snails in controlled choice feeding trials with live snails (Fig.4). This preference for J-snails could be due to differences in the allometry of shell strength between S-snails and J-snails (estimated here as mean shell ridge thickness, Fig.2B; Cotton et al. 2004). However, the importance of shell strength for shell-entry predators such as conchs is not clear. Further, crown conchs eat similar numbers of (or even more) S-snails than J-snails when the snails are dead (Fig.4), suggesting that shell strength is not an important determinant of consumption. Collectively, our results support the conjecture that reduced predator avoidance responses of J-snails to conchs contribute to the higher consumption of live J-snails, although we cannot rule out an effect of temporal variation, as live and dead snail trials were conducted at different times.

Snail trait variation based on host plant species had a “carry-over” effect (Van Allen and Rudolf 2013) on tritrophic interactions that influenced marsh plant species performance and relative abundance over seasonal time scales. Snail trait variation was amplified in the presence of predators compared to their absence (Araujo et al. 2011), at least in terms of the effects on *Juncus* stem height (Fig.7) and aboveground biomass (Fig. S1). For stem height, which snail type had the most negative effect on *Juncus* depended on predator presence or absence in the *Juncus*-only community (Fig.7). In addition, in the mixed plant community, J-snails and S-snails had similar effects on *Juncus* in the absence of predators (Fig.7B), but J-snails caused a greater reduction in height than S-snails when predators were present (Fig.7A). The effects of snail trait differences on *Juncus* aboveground biomass were similarly amplified by predators in the *Juncus*-only community (Fig. S1b-c). Thus, predicting the effects of top predators in this system may not be possible just based on predator presence/absence, but instead will require information regarding snail traits and plant species composition (c.f., Urban 2013). Because we standardized shell length between snail types in our mesocosm experiment, it likely provides a conservative estimate of the effects of intraspecific trait variation. Snail body size influences the strength of snail impacts on plants, with larger snails inflicting greater leaf damage than smaller snails (Atkins et al. 2015). Thus, we predict that differences in effect size between snail types may be even greater in natural assemblages than observed in our mesocosm experiment.

The generally negative effects of snails on *Juncus* in our experiment were notable and somewhat surprising. Snails readily climb on *Juncus*, likely due to its enhanced refuge from predation (Hughes 2012); in fact, more snails on average were found climbing on *Juncus* than on *Spartina* in our experiment. Although the exact mechanism is unclear, our data point to a negative effect of this refuge use on *Juncus* (c.f., Sotka et al. 1999). We have observed similar negative effects of *Littoraria* on *Juncus* in field experiments using natural assemblages of *Juncus* (Hughes 2012; Zerebecki unpublished data), suggesting that the poor performance of *Juncus* in this experiment was not merely an artifact of our experimental mesocosms. While *Littoraria* will consume *Juncus* litter (Zimmer et al. 2004), they are not known to consume standing live or dead stems, and we did not observe any visible grazing scars in our mesocosm experiment. Even when structural defenses of *Spartina* and *Juncus* were eliminated in our agar-based feeding trial, few snails consumed *Juncus* tissue (Fig.3), perhaps due to its lower nutrient content (Pennings et al. 1998). However, stable isotope analysis of S-snails and J-snails collected from mixed plant assemblages identified significant differences in isotopic composition between snail types (Fig.3B), indicating that the primary food source(s) of each snail type may differ consistently over weeks to months. These data also suggest that *Juncus* may be used both as a refuge and food source by J-snails: The δ^13^C of C3 plants like *Juncus* is distinct from that of C4 plants like *Spartina* (−26‰ compared to −13‰; Sullivan and Moncreiff 1990), and J-snails have a more negative C isotopic signature (mean = −18.2‰) than S-snails (mean = −16.2‰). Consequently, while both snail types may prefer to consume *Spartina* in a controlled feeding trial in the absence of predation, trophic specialization may be partially driving microgeographic divergence in this population.

Phenotypic differences within species over very small spatial scales are often ascribed to ecological processes such as plasticity (Richardson et al. 2014), and plasticity in morphological and behavioral variation has commonly been documented in gastropod species (e.g., Trussell 1996; Trussell et al. 2003). *Littoraria* has a planktonic larval stage (30 days in the plankton; Diaz-Ferguson et al. 2010), increasing the likelihood that subsequent generations will encounter either host plant species. Thus, plasticity via preference induction, or the ability of experience with particular stimuli (here, a given host plant) to increase subsequent preferences for those same stimuli (Dethier 1982; Davis and Stamps 2004), could contribute to the carry-over effects (Van Allen and Rudolf 2013) of snail host plant documented here. Preference induction tends to be common in organisms that use the same plants for habitat and food (Jermy 1987; Hultgren and Stachowicz 2010). It is unclear whether host plant has strong and persistent effects on later habitat and/or food preferences beyond the time scale examined in this study, or whether sustained experience with alternative stimuli could reverse the effects of previous induction (Davis and Stamps 2004; Hoverman and Relyea 2007).

*Littoraria* populations along the US Atlantic coastline showed little phylogeographic structure in a prior study (Diaz-Ferguson et al. 2010), and our own preliminary data show little sequence divergence between S-snails and J-snails (Hanley unpublished data). However, we currently cannot rule out the role of microgeographic adaptation in this system. Such adaptation occurs across a range of habitats and taxa, even in the presence of widespread dispersal and an absence of variation at neutral genetic markers (Conover et al. 2006, Richardson et al. 2014). Habitat selection, the process common to phytophagous insects wherein genotypes display preferences for different habitats over fine spatial scales (Richardson et al. 2014), is one potential mechanism that could be operating in this system. In addition, preferential consumption of J-snails by conchs, particularly in *Spartina*-only habitats, may promote differentiation across host plant species by limiting the ability of J-snails to mate with S-snails and/or contribute to the population (c.f., Nosil 2004). Regardless of the specific ecological or evolutionary mechanism(s) contributing to trait divergence in J-snails and S-snails, it is clear that these behavioral and morphological differences are ecologically important and deserve further attention (Dall et al. 2012).

Given that host plant species is the apparent driver of consumer trait variation in this species, it is interesting that the relative importance of snail type compared to predator treatment differs for *Juncus* and *Spartina*. For instance, the effects of snails on *Spartina*, their preferred food source, are generally consistent across snail types both in the presence and absence of a predator. Although snail types display clear differences in feeding rate (Fig.3A) and climbing behavior (Fig.5), which likely contribute to differential effects on *Spartina* stem height (Fig.7C), the resultant effects of this trait variation on *Spartina* density and biomass are negligible compared to the cascading effects of a top predator in this system (Fig. S2). This positive effect of predators on *Spartina* suggests that changes in snail foraging rate due to direct or indirect predator effects are consistent across snail types (Kimbro 2012; Toscano and Griffen 2014). In contrast, the combination of snail trait variation and predator presence/absence strongly influenced *Juncus* height, density, and biomass. These differences in the relative importance of consumer trait variation and top predator presence may be due to variation in the relative importance of *Juncus* and *Spartina* as a source of refuge versus a source of food for *Littoraria*. That *Juncus* and *Spartina* serve different functions for snails is supported by the finding that consumer trait variation differentially affected the plant community more often in the single-species than mixed plant communities (c.f., Edwards et al. 2010; Hughes 2012). Thus, consumer intraspecific variation has the potential to interact with plant species diversity to alter species interactions and trophic dynamics, in turn impacting interaction strength and ultimately food web stability (Bolnick et al. 2011; Gibert and Brassil 2014).

## Data Accessibility

Data available from the Dryad Digital Repository: http://dx.doi.org/10.5061/dryad.3q9k0

## Supporting Information

**Figure S1.** Effects of plant community composition, predator presence, and snail type on *Juncus* density and aboveground biomass. **Figure S2**. Effects of predator presence and plant community composition on change in live plant stems, final aboveground plant biomass, and *Spartina* belowground biomass. **Figure S3**. Number of snails of each type per *Spartina* stem at high tide in the presence and absence of a predator in a 12-week mesocosm experiment.
