# Supplementary material for: Consumer trait variation influences tritrophic interactions in salt marsh communities
Source: Ecol Evol. 2015 Jun 17;5(13):2659–72. doi: 10.1002/ece3.1564 (PMC4523361; doi:10.1002/ece3.1564)

**Online Supporting Information**

FIGURE S1. Effects of plant community composition, predator presence, and snail type on (a) the change in *Juncus* density and (b,c) *Juncus* aboveground biomass. (a) Fewer *Juncus* stems died in the mixed plant treatment than the *Juncus*-only treatment, and this effect was consistent across predator treatment and snail type. (b) When predators were present, *Juncus* aboveground biomass was greater with J-snails than S-snails in the *Juncus*-only community, but there were no differences by snail type in the mixed community. (c) When predators were absent, there were no differences by snail type in either plant community. Letters indicate significant differences at P<0.05 based on Tukey's post hoc tests. Bars represent means + 1SE.


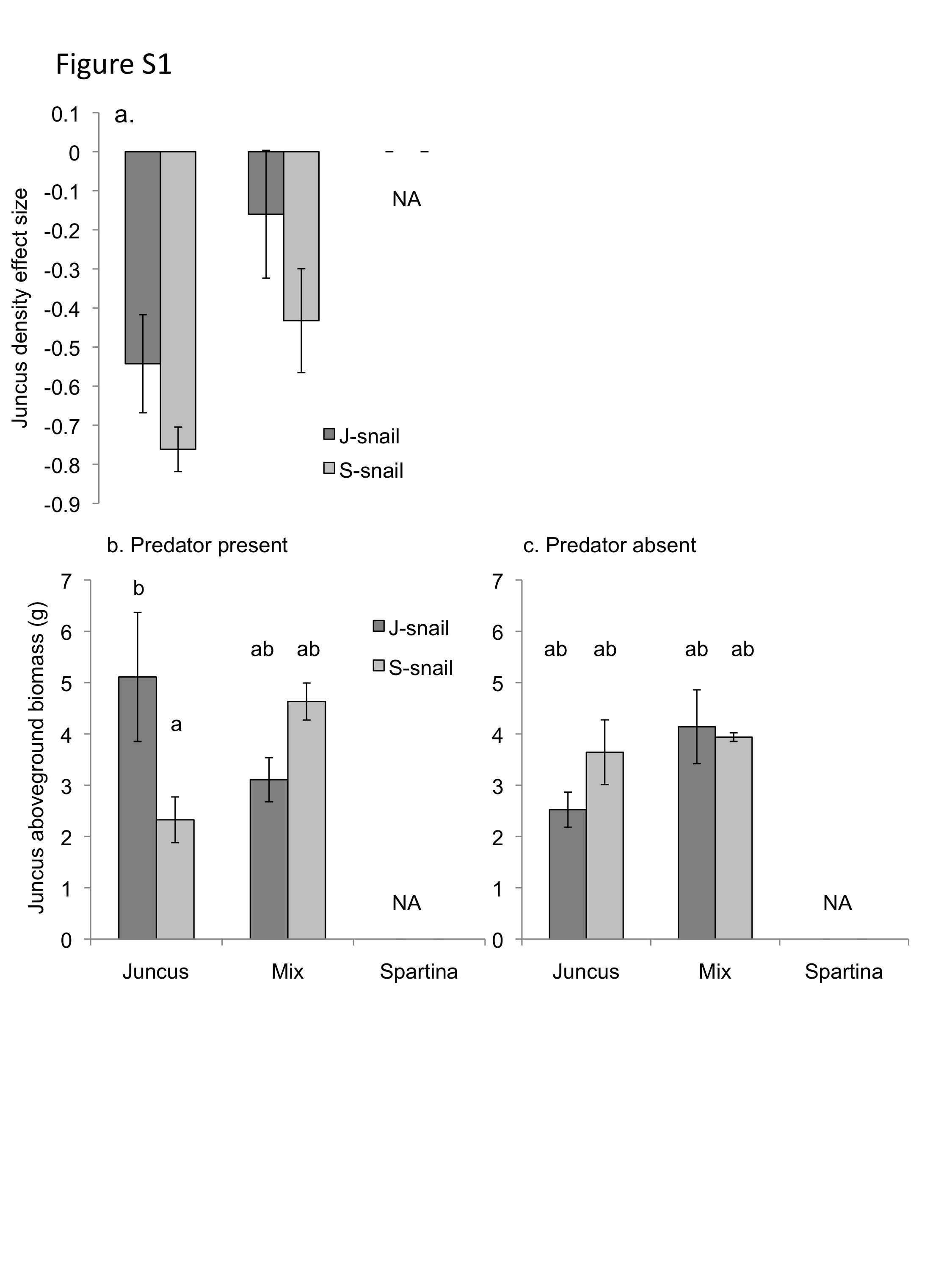
FIGURE S2. Effects of predator presence and plant community composition on (a) change in live plant stems, (b) final aboveground plant biomass, and (c) *Spartina* belowground biomass. For all three measures, predator presence had a positive effect on plant responses. (a,b) There was a greater loss of live stems and reduced total aboveground biomass in *Juncus*-only communities compared to mixed or *Spartina*-only communities. (c) *Spartina* aboveground biomass was higher in *Spartina*-only than mixed plant communities. There was also a marginal positive effect of predator presence on *Spartina* aboveground biomass. Letters indicate significant differences at P< 0.05 based on Tukey's post hoc tests. Bars represent means + 1SE.


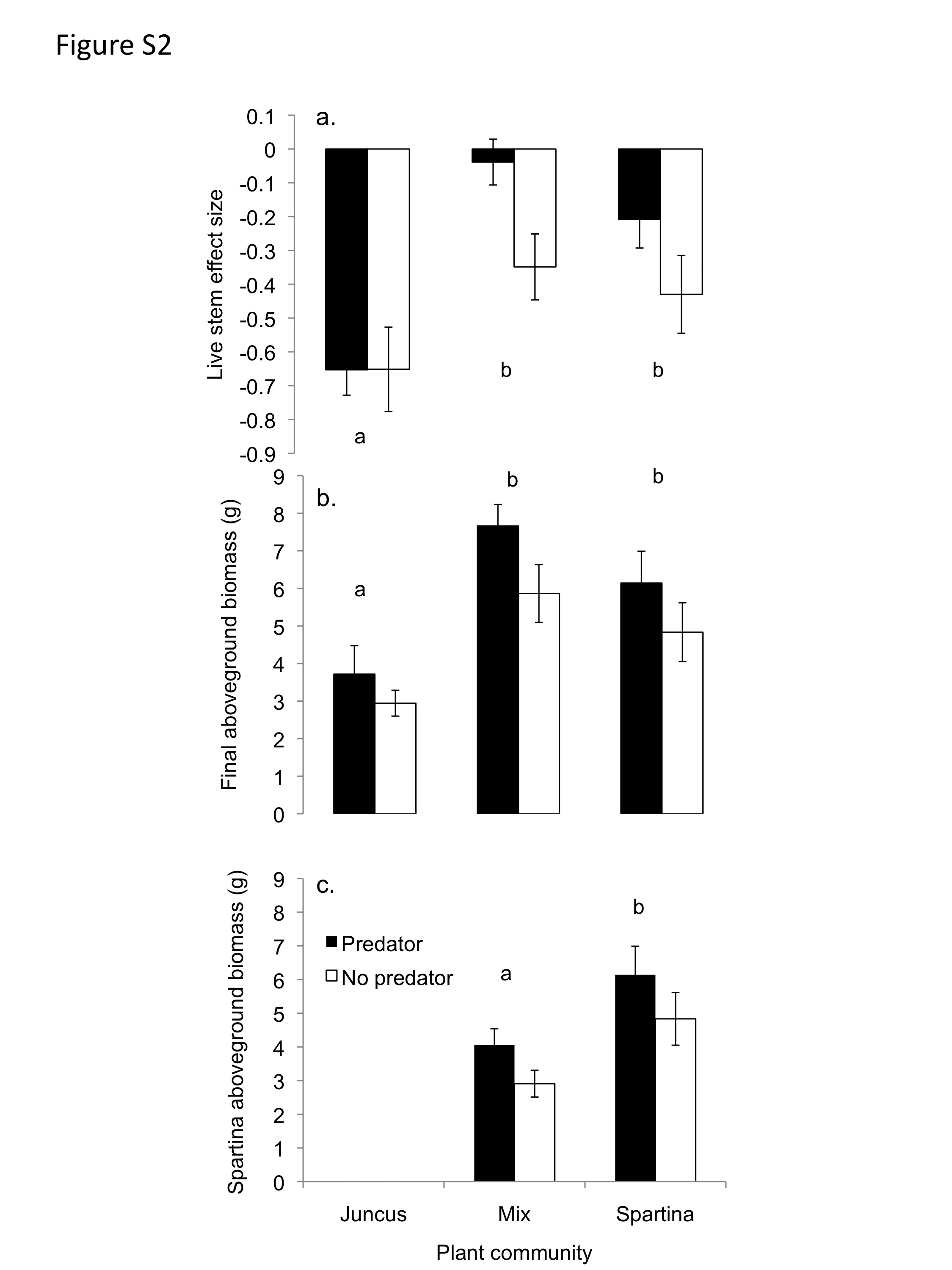
FIGURE S3. Number of snails of each type per *Spartina* stem at high tide in the (a) presence and (b) absence of a predator in a 12-week mesocosm experiment. The number of snails per *Spartina* stem was equivalently low across snail types and plant composition in the presence of a predator. In contrast, more J-snails climbed on *Spartina* in the *Spartina*-only treatment when predators were absent. Letters indicate significant differences at P< 0.05 based on Tukey's post hoc tests. Bars represent means + 1SE.


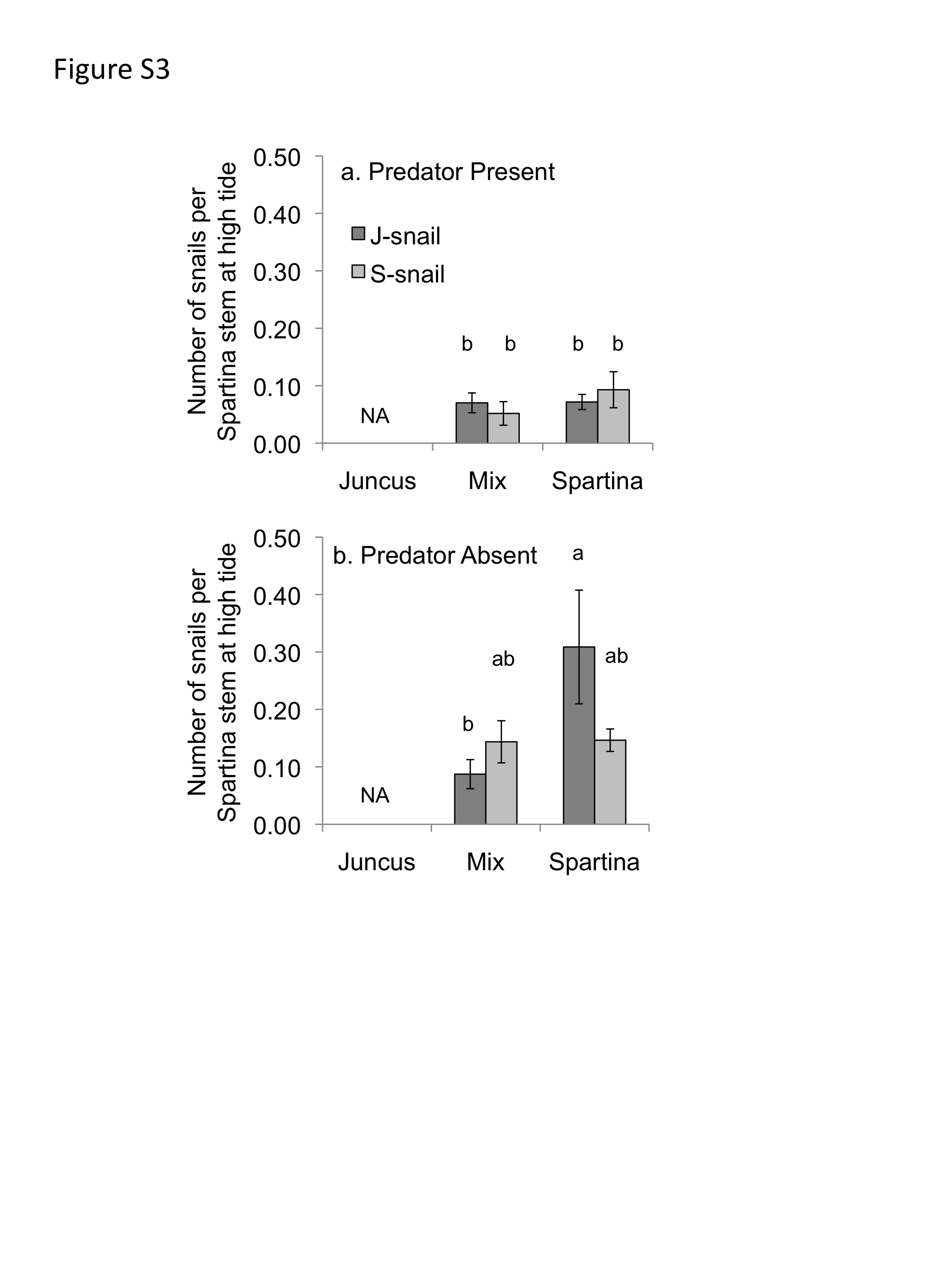

Supplement: Supplementary file 1 [file ece30005-2659-sd1.docx]
